# Age-related changes in static and dynamic postural balance performance

**DOI:** 10.3389/fnagi.2026.1759879

**Published:** 2026-03-04

**Authors:** A. Rizzato, M. Bozzato, A. Paoli, S. Faggian, G. Marcolin

**Affiliations:** 1 Department of Biomedical Sciences, University of Padova, Padua, Italy; 2 Department of Industrial Engineering, University of Padova, Padua, Italy

**Keywords:** aging, balance, center of pressure, fall risk, perturbations, postural control, sample entropy

## Abstract

**Background:**

Age-related changes in the neuromuscular and sensory systems compromise the control of balance and stability. Static balance assessments may overlook deficits that appear when coping with unexpected perturbations. This cross-sectional study aimed to compare static and dynamic balance performance in younger and older adults to assess age-related differences in postural control between the two age groups.

**Methods:**

Sixty-nine younger adults (24.3 ± 0.4 years) and sixty-one older adults (72.1 ± 0.6 years) performed balance assessments under static and dynamic conditions on a force platform. Center of pressure (CoP) was calculated during quiet standing for static balance and during an unexpected perturbation of the base of support for dynamic balance. In the perturbation-based task, the following CoP-related parameters were analyzed within a 2.5-s window from perturbation onset: displacement (Area95D), Mean VelocityD, anterior–posterior first peak (FP), post-perturbation variability (PPV), and maximal oscillations (ΔCoPMax). Sample Entropy (SampEn X and Y) was computed to infer the automaticity of postural control.

**Results:**

In the static test, balance performance did not differ between younger and older adults, although older adults exhibited reduced efficiency (*p* < 0.05). Dynamic balance showed age-related differences, with older adults highlighting larger Area95D (*p* < 0.001), higher Mean VelocityD (*p* < 0.001), and greater FP (*p* < 0.05). SampEn X did not differ between groups, whereas SampEn Y was lower in older adults (*p* < 0.001).

**Conclusion:**

Age-related changes in balance control are task dependent. Older adults preserved static balance performance but demonstrated impaired reactive balance responses in dynamic tasks. Furthermore, static and dynamic balance rely on distinct control mechanisms, highlighting the need for separate assessments.

## Introduction

Postural balance is a complex motor function crucial for maintaining stability during everyday activities and preventing falls (Horak, 2006). Aging is known to affect multiple components of balance, including sensory integration, neuromuscular coordination, and motor response speed (Wang et al., 2024). Such age-related deteriorations can increase the risk of falls, which represent a major health concern for the elderly population (Xu et al., 2022). Effective balance performance demands the ability to adapt muscular responses to changing balance threats (Lin and Woollacott, 2002), which can vary in magnitude and direction. Maintaining this adaptive capacity is essential to effectively respond to balance challenges and reduce fall risk in older adults.

Conventional balance assessments often focus on static postural control during quiet standing (Nardone et al., 1997; Pajala et al., 2008; Marinho-Buzelli et al., 2017), measuring center of pressure (CoP) displacement to quantify balance performance objectively (Rizzato et al., 2023a). However, static tests alone cannot fully capture the adaptive components of postural control (Petró et al., 2017) and may fail to distinguish between individuals with similar balance performance but different functional capacities (Rizzato et al., 2025b). To address these limitations, dynamic balance assessments have been increasingly employed to evaluate postural control under more functionally relevant conditions (Mancini and Horak, 2010; Petró et al., 2017). However, dynamic balance tests often involve self-initiated movements, providing important but limited information about an individual’s overall balance ability. Indeed, self-initiated tasks (e.g., sit-to-stand or the timed-up-and-go test) primarily assess controlled movements performed within an individual’s established limits of stability (Podsiadlo and Richardson, 1991; Pai et al., 2010; Muehlbauer et al., 2012). As such, they are typically performed under predictable and controlled conditions, allowing for anticipatory postural adjustments and minimizing the need for rapid, reactive responses to unexpected perturbations.

Real-life scenarios frequently involve unexpected perturbations, such as slips, trips, or sudden changes in the support surface, that require rapid and efficient reactive postural adjustments to prevent loss of balance and potential falls (Ghulyan et al., 2005; Zemková et al., 2016). These unanticipated events place higher demands on the neuromuscular system, challenging the ability to generate timely and coordinated responses without prior preparation. To better understand reactive components of dynamic balance performance, perturbation-based protocols have been developed (Chen et al., 2014; Inkol and Vallis, 2019; Bosquée et al., 2021). This approach enables controlled delivery of external perturbations to the base of support, thereby eliciting reactive, involuntary balance responses.

Despite the importance of reactive postural control, most investigations in older adults have focused on perturbation responses during walking (Bhatt and Pai, 2009; Oliveira et al., 2012), rather than during standing tasks, which may involve different demands on postural regulation. Moreover, few studies have directly compared younger and older adults under both static and perturbation-based conditions, and those have often yielded conflicting results, limiting the understanding of age-related differences in balance control strategies. For instance, Gu et al. (1996) found that healthy older adults did not exhibit substantial differences from young adults in response to modest unexpected perturbations, suggesting that age-related changes in reactive balance control may be trivial and context dependent. Conversely, Bierbaum et al. (2010) found age-related deficits in dynamic stability in older adults, evaluated over an unexpected-perturbed walking approach. Similarly, Baloh et al. (1994) reported significantly greater sway velocity in older adults compared to younger individuals, with more pronounced differences observed during dynamic (i.e., perturbation of the base of support) than static posturography. In another example given by Nakamura and colleagues, a greater anterior–posterior CoP displacement was reported in elderly people compared to a younger counterpart in unexpected forward perturbation of the base of support (Nakamura et al., 2001). However, these last experimental setups (Baloh et al., 1994; Nakamura et al., 2001) involved small-magnitude perturbations that were delivered in a predictable manner across trials, allowing participants to anticipate the disturbance. Therefore, reactive responses to unexpected perturbations, particularly in aging populations, remain insufficiently investigated through objective methodologies. This gap highlights the need for more comprehensive approaches that integrate both static and dynamic assessments, since relying solely on static measures may overlook individuals with preserved static balance but reduced responsiveness to real-life external perturbations.

In addition to conventional measures of postural sway, nonlinear analyses such as Sample Entropy (SampEn) can provide valuable insights into the underlying control mechanisms of balance. SampEn quantifies the regularity and predictability of center of pressure (CoP) displacement, with higher values indicating higher irregularity and thus, more automated control strategies (Donker et al., 2007; Roerdink et al., 2011). Age-related changes in sensory integration and neuromuscular control may alter the complexity of postural regulation, leading to reduced adaptability and increased rigidity in balance control systems (Manor et al., 2010). Evaluating SampEn during balance tasks may therefore reveal subtle differences in balance control strategies between younger and older adults, providing complementary information to traditional CoP-based metrics.

Given the multifactorial processes underlying fall prevention, understanding whether both static and dynamic balance assessments are required for accurate evaluation and targeted intervention represents a key question. Therefore, this study aims to provide a comparative analysis of static and dynamic balance performance in older and younger adults using objective force platform assessments during quiet standing and perturbation-based tasks. We hypothesized that, relative to younger controls, older adults would demonstrate greater impairments in balance performance during dynamic, perturbation-based task than during static task. Moreover, we expected that older adults would rely more heavily on voluntary and less adaptable postural control mechanisms compared with younger adults. Additionally, the study examined the relationship between static and dynamic balance performance in younger and older adults. Considering that balance control has been shown to be highly task-specific, we hypothesized that performance in static and dynamic conditions would not be correlated across age groups.

## Methods

### Subjects

A total of 130 subjects were recruited for the study, divided into two groups: younger adults (YA) and older adults (OA). The YA group comprised 69 subjects (*F* = 33; mean ± standard error (SE): age 24.29 ± 0.39 years; height 1.72 ± 0.01 m; body mass 65.59 ± 1.48 kg), while the OA group consisted of 61 subjects (*F* = 33; mean ± SE: age 72.13 ± 0.64 years; height 1.65 ± 0.01 m; body mass 69.52 ± 1.62 Kg). Before enrollment, all subjects were screened via a telephone interview to determine their eligibility. The YA group met the following inclusion criteria: (i) age between 18 and 35 years and (ii) no participation in professional or national-level sports competitions. The inclusion criteria for the OA group were: (i) age between 65 and 85 years and (ii) autonomy in activities of daily living.

Additional factors relevant to balance interpretation in older adults were also considered. All older adults in the study were independent in their activities of daily living and had no history of falls. Vision impairments, if present, were corrected using glasses. No participants in the younger adult group engaged in competitive or organized sports activities. Exclusion criteria for both groups were: (i) uncorrected visual impairments, (ii) psychiatric or neurological disorders, (iii) regular use of medications that could interfere with cognitive function (e.g., antidepressants, antipsychotics, anxiolytics), (iv) orthopedic injuries in the last 3 months, and (v) previous experience with unstable devices. Written informed consent was obtained from all participants after they were fully briefed on the study’s procedures.

### Study design

The experimental protocol received approval from the Human Ethical Committee of the Department of Biomedical Sciences at the University of Padova (n° HEC-DSB/05–21) and adhered to the principles outlined in the Declaration of Helsinki. The study was designed and reported following the STROBE guidelines for cross-sectional observational studies. The static balance assessment was conducted through a standardized bipedal balance test, while dynamic balance control was evaluated using a perturbation-based test. For both static and dynamic balance tests, a 60 cm × 40 cm force plate (AMTI BP400600, Watertown, MA, USA) was used. Data were acquired at a sampling rate of 200 Hz, and the CoP trajectory was computed using the Balance Clinic 1.4.2 software (AMTI, Watertown, MA, USA). Prior to each assessment, a familiarization session was conducted to ensure participants understood the procedure and were able to perform the tasks correctly.

### Measurements

#### Static balance test

During the bipedal static balance test, subjects were instructed to maintain an upright stance with their arms positioned alongside their bodies. They were required to focus on a narrow red line, vertically positioned on a white wall placed 80 cm in front of them. Foot placement on the force platform was standardized using a V-shaped frame, ensuring a 7 cm separation between the heels and a 30° angular alignment between the toes, in accordance with guidelines from the International Society of Posturography (Kapteyn et al., 1983). A total of three trials were performed, each lasting 30 s, with a 60-s rest interval between trials.

#### Dynamic balance: perturbation-based test

**Figure 1:**
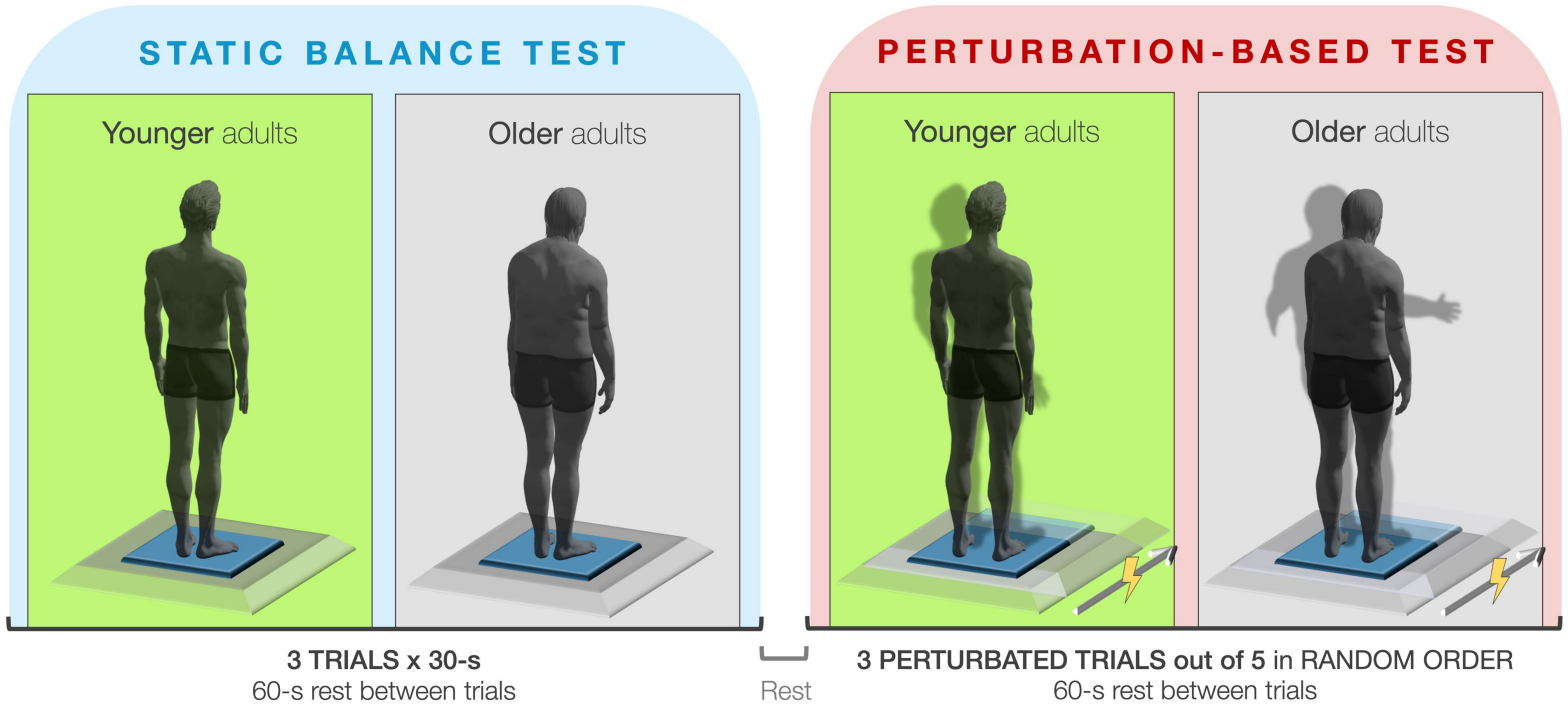
Graphical representation of the experimental design.

Dynamic balance was evaluated using a perturbation-based protocol employing a motorized movable platform (EnginLAB s.r.l., Padova, Italy), previously described in detail (Rizzato et al., 2023b). The system, controlled by a dedicated software (RTC-9000, EnginLAB s.r.l., Padova, Italy), could deliver unexpected perturbations to the base of support by modulating both the displacement amplitude and the velocity profile (i.e., ramp rate) of the platform. In this experimental setup, the platform was programmed to translate 50 mm at a ramp rate of 100 mm/s. The perturbation was applied in the anterior direction relative to the subject’s standing posture (i.e., forward). The force plate was screwed on top of the movable platform. Synchronization between the force plate and the movable platform was achieved using an external trigger box. Each trial lasted 30 s, during which a single perturbation was delivered at a random time point between the 5th and 25th second. To further minimize participant anticipation, two of the five administered trials did not include any perturbation and were randomly interspersed throughout the session. This randomization of both perturbation timing and trial order effectively reduced the possibility of participants predicting the perturbation and preventing any learning effects across trials. Additionally, a silent mouse was used to eliminate any potential auditory or tactile cues from the operator, ensuring that participants could not anticipate the perturbation through external signals. Participants wore a safety harness connected to an overhead support structure to prevent them from falling. The harness system was designed not to interfere with natural posture or reactive movements in response to the perturbation. During each trial, subjects stood quietly on the platform with arms relaxed at their sides and maintained gaze on a fixed visual target positioned 80 cm ahead at eye level. The experimental design is presented in Figure 1.

### Data analysis

#### Static balance test

From the CoP trajectory, two parameters were calculated: Area95, defined as the area (expressed in cm^2^) of the 95% confidence ellipse encompassing the CoP data points, and Mean Velocity, calculated as the total path length divided by the trial duration and expressed in cm⋅s^−1^. Area95 represents an overall index of balance performance: the smaller Area95, the better the performance. Mean Velocity represents the efficiency of postural control: the lower the CoP velocity, the more the efficiency (Paillard and Noé, 2015). Each parameter was computed for all trials and subsequently averaged across the three static balance tests.

#### Dynamic balance: perturbation-based test

A set of CoP-based variables was computed within a 2.5-s analysis window (Rizzato et al., 2023b), starting from the perturbation point (PP), defined as the onset of the platform displacement. Within the entire 2.5-s interval, Area95D was calculated as the area in cm^2^ of the 95% confidence ellipse of the CoP trajectory, while Mean VelocityD referred to the mean CoP velocity and was expressed in cm/s. In addition to these global measures, the first peak (FP) was derived from the anterior–posterior (AP) CoP displacement as the difference between the maximum AP displacement reached after the perturbation onset and the baseline mean CoP value prior to PP. Moreover, the maximum oscillation amplitude (ΔCoPMax), defined as the difference between the maximum CoP displacements in the positive and negative directions, was calculated separately for both the anterior–posterior (ΔCoPMax_AP) and medio-lateral (ΔCoPMax_ML) directions. The FP and ΔCoPMax parameters primarily reflect the initial feet-in-place postural adjustments elicited by the perturbation. Lower values in these measures are indicative of more effective and timely postural responses (Rizzato et al., 2023b). Additionally, post-perturbation variability (PPV) was computed as the standard deviation (SD) of the CoP displacement within the 2.5-s window following the perturbation onset. This parameter reflects the subject’s ability to regain postural stability in response to the perturbation. For all dynamic variables, values were computed separately for each perturbed trial and then averaged across the three perturbed trials for each participant. A lower PPV denotes a quicker and more efficient return to quiet standing. The analysis tool was developed with MATLAB R2024a (TheMathWorks, Inc., Massachusetts).

#### Sample entropy

SampEn was computed in both the anterior–posterior and medio-lateral CoP components —referred to as SampEn Y and SampEn X, respectively—for the static balance tests. The calculation followed the procedure described by Ramdani and colleagues (Ramdani et al., 2009), previously adopted elsewhere (Rizzato et al., 2018). SampEn was computed according to the following formulation:

SampEn(m,r,N)=−lnCP(m,r)

where *N* denotes the total number of CoP data points, and *CP(m, r)* represents the conditional probability that two sequences of length *m* that match within a tolerance *r* will also match at the next point. This approach quantifies the regularity and complexity of postural sway, with lower SampEn values reflecting greater signal regularity. In the present analysis, the embedding dimension was set to *m = 3* and the tolerance threshold to *r = 0.35*, consistent with the methodological framework reported by Ramdani et al. (2009). All computations were carried out in MATLAB R2024a (TheMathWorks, Inc., Massachusetts).

### Statistical analysis

The primary outcome of this cross-sectional study was balance performance (i.e., Area95 and Area95D). An *a priori* power analysis for an unpaired two-tailed *T*-test was conducted on the primary outcome (G*Power 3.1.9.4, Germany). The input parameters were an alpha error probability of 0.05, an effect size of d = 0.5, and a statistical power of 0.8. The analysis indicated a total required sample size of 128 participants.

Data were screened for outliers using the interquartile range (IQR) criterion (Mosteller et al., 1977), independently for each age group. Subjects identified as outliers on any variable within static balance, dynamic balance, or SampEn, were excluded only from the corresponding analyses. Thereafter, the Shapiro–Wilk test was used to check the normality of data distribution in both age groups. Independent samples T-tests were applied to detect differences of static and dynamic parameters between the two age groups for each variable. In cases where the assumption of normality was violated, group comparisons were performed using the non-parametric Mann–Whitney *U* test. False discovery rate (FDR) *p*-value correction for multiple comparisons was applied using the Benjamini-Hochberg procedure (Benjamini and Hochberg, 1995), separately for static balance, dynamic balance, and SampEn. All reported *p*-values are FDR-adjusted. For the *T*-tests, the effect size (ES) was given by Cohen’s d and interpreted as follows: small (0.2 ≤ d < 0.5), medium (0.5 ≤ d < 0.8), and large (*d* ≥ 0.8) (Cohen, 1988). For the Mann–Whitney *U* test, the ES was given by the rank biserial correlation. Spearman’s correlation was used to assess the association between static and dynamic balance due to the violation of normality assumption for those variables. Correlation was tested both within each group and across the entire sample. Ninety-five percent confidence intervals (95% CI) are reported for the correlation coefficients. The level of significance was set to *p* < 0.05. The statistical analysis was performed with JASP software version 0.19.3 (University of Amsterdam, Amsterdam, The Netherlands). Data are presented as mean ± standard error (SE).

## Results

Following the removal of outliers, 7 subjects were excluded from the static condition (i.e., YA: 3; OA: 4), 17 from the dynamic condition (i.e., YA: 10; OA: 7), and 2 from SampEn analysis (i.e., OA: 2).

### Static balance

**Figure 2:**
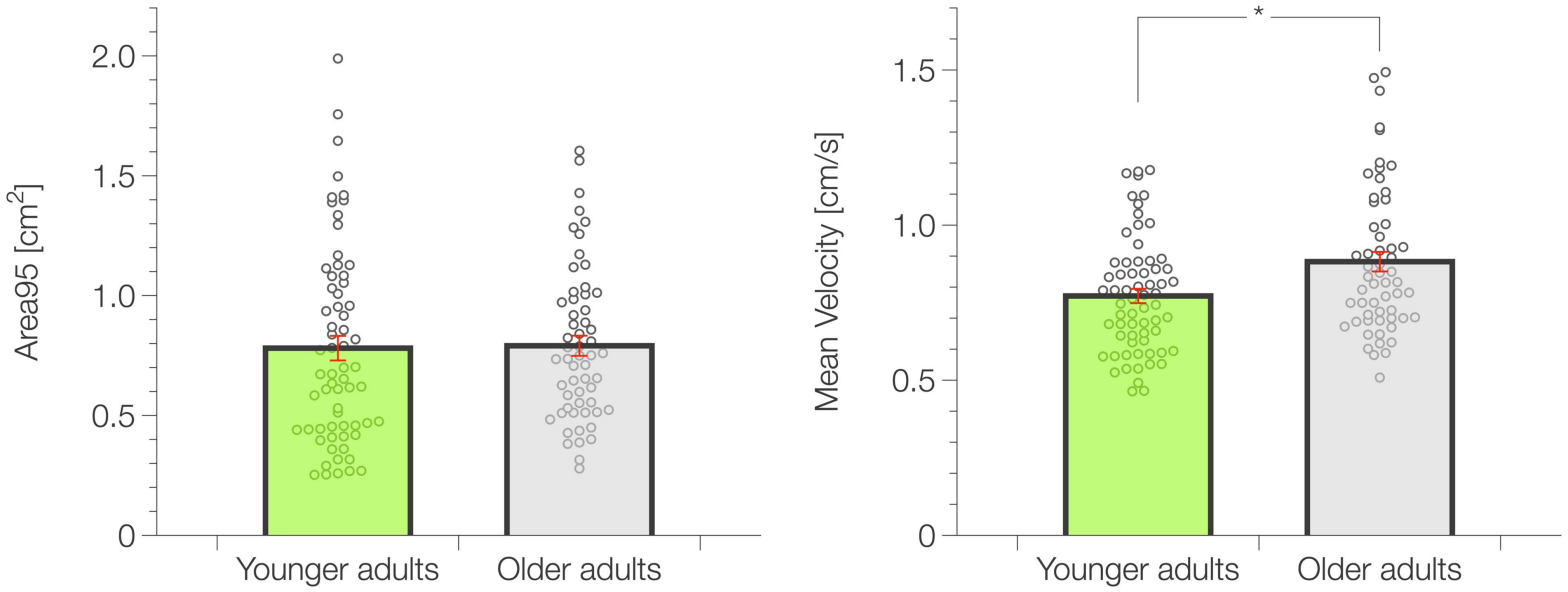
Results of the static balance test. Data are presented as mean ± standard error (SE). *p < 0.05.

The overall static balance performance represented by the Area95 did not show statistically significant differences (*p* = 0.390; ES = 0.090) between YA (0.78 ± 0.05 cm^2^) and OA (0.80 ± 0.04 cm^2^). Conversely, the postural control efficiency, expressed by the Mean Velocity, was significantly higher (*p* = 0.022; ES = 0.267) in the YA group (0.77 ± 0.02 cm⋅s^−1^) than OA (0.88 ± 0.03 cm⋅s^−1^). A graphical representation of the static balance results and data distribution is represented in Figure 2.

### Dynamic balance

**Figure 3:**
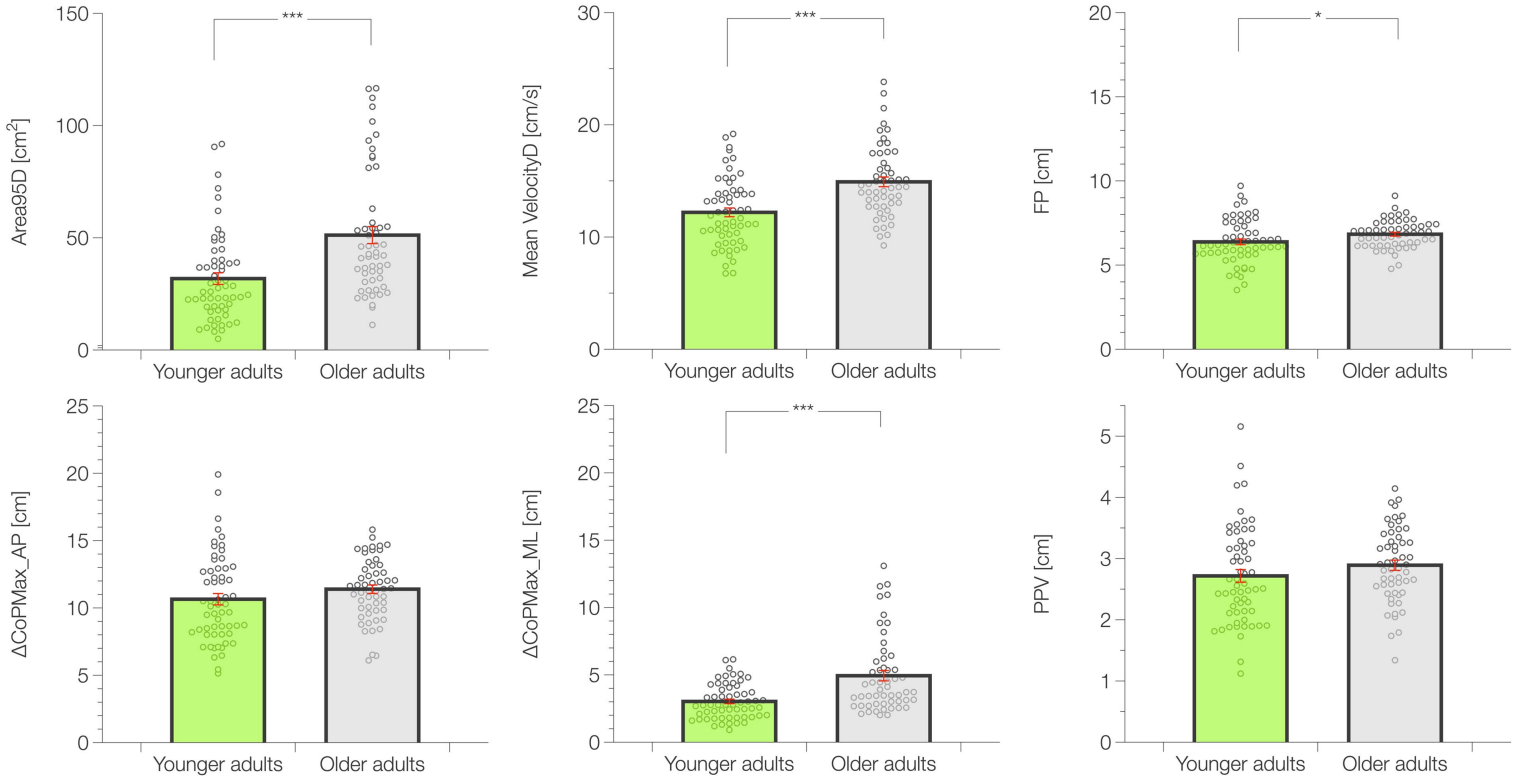
Results of the dynamic balance test. Data are presented as mean ± standard error (SE). *p < 0.05, ***p < 0.001.

In the 2.5-s time window following the unexpected perturbation, Area95D was different (*p* < 0.001; ES = 0.476), being lower in YA (31.81 ± 2.59 cm^2^) compared to OA (51.17 ± 3.79 cm^2^). Mean VelocityD as well was significantly higher in OA (*p* < 0.001; ES = 0.465; YA = 12.20 ± 0.39 cm⋅s^−1^; OA = 14.91 ± 0.43 cm⋅s^−1^). The FP showed the same trend, with higher values recorded in OA (*p* = 0.045; ES = 0.413; YA = 6.38 ± 0.17 cm; OA = 6.83 ± 0.11 cm). As per the maximal oscillations, in the anterior posterior axis (ΔCoPMax_AP) YA and OA showed similar results (*p* = 0.198; ES = 0.263; YA = 10.64 ± 0.42 cm; OA = 11.39 ± 0.31 cm); in the medio-lateral axis (ΔCoPMax_ML) instead YA revealed better postural control than OA (*p* < 0.001; ES = 0.458; YA = 3.03 ± 0.17 cm; OA = 4.94 ± 0.39 cm). Lastly, PPV did not differ in the two age groups (*p* = 0.198; ES = 0.245; YA = 2.72 ± 0.10 cm; OA = 2.89 ± 0.08 cm). A graphical representation of the dynamic balance results and data distribution is available in Figure 3.

### Sample entropy

**Figure 4:**
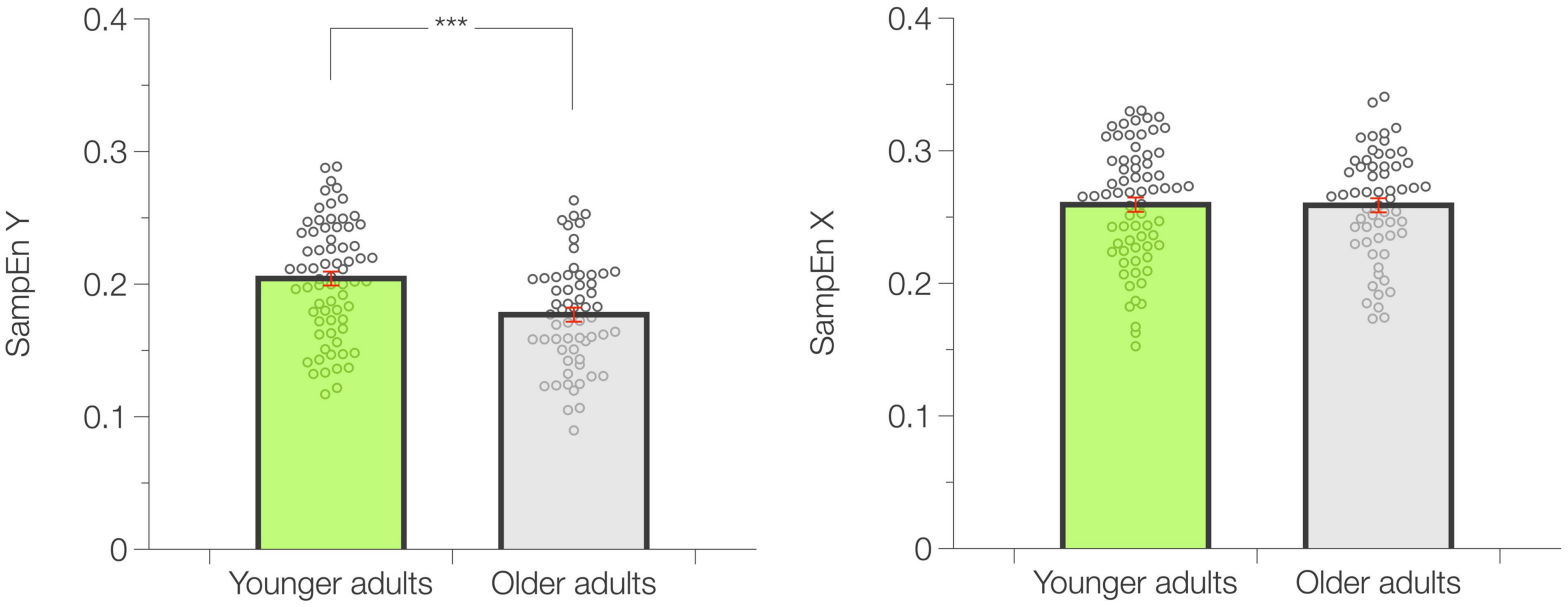
Results of the sample entropy analysis. Data are presented as mean ± standard error (SE). ***p < 0.001.

SampEn X resulted similar (*p* = 0.871; ES = −0.017) in YA (0.26 ± 0.01) and OA (0.26 ± 0.01). However, YA showed higher values of SampEn Y compared to OA (*p* < 0.001; ES = −0.645; YA = 0.20 ± 0.01; OA = 0.18 ± 0.01). SampEn results and data distribution are presented in Figure 4.

### Static and dynamic balance correlation

**Figure 5:**
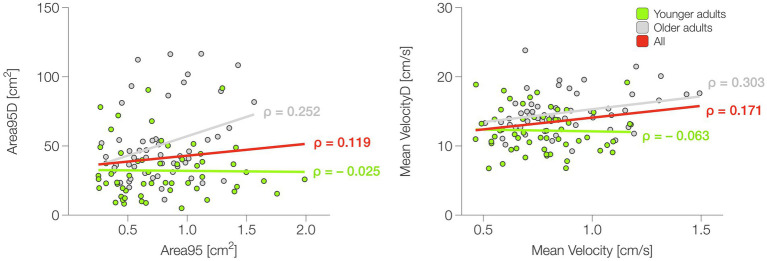
Results of the correlation between static and dynamic balance. ρ, Spearman’s rank correlation coefficient.

For both groups, the correlation between static (Area95) and dynamic (Area95D) balance performance was not significant [YA: Spearman’s *ρ* = −0.025; *p* = 0. 851; 95% CI (−0.237, 0.284); OA: Spearman’s ρ = 0.252; *p* = 0. 072; CI (−0.022, 0.491)]. The same results were obtained considering the whole sample [Spearman’s ρ = 0. 119, *p* = 0.216; CI (−0.070, 0.300)]. The correlation between static (Mean Velocity) and dynamic (Mean VelocityD) balance efficiency was not significant in YA [Spearman’s ρ = −0.063; *p* = 0.638; 95% CI (−0.319, 0.200)], whereas a low significant correlation was found in OA [Spearman’s ρ = 0.303; *p* = 0.029; 95% CI (0.033, 0.532)]. Across the whole sample there was no significant correlation [Spearman’s ρ = 0.171; *p* = 0.076; CI (−0.018, 0.348)]. Correlations are graphically presented in Figure 5.

## Discussion

### Balance performance and postural control mechanisms

The primary aim of this study was to provide a comparative analysis of static and dynamic balance performance in older and younger adults, based on objective CoP-related parameters calculated during both quiet standing and perturbation-based tasks. The main findings highlighted reduced postural control efficiency in older adults when coping with both static and dynamic balance tasks. Surprisingly, balance performance differed only in response to the perturbation-based task, with younger adults showing superior performance.

These findings indicate that aging may negatively affect the reactive components of postural response to an external perturbation, while preserving static balance performance. The deficits observed in dynamic balance among older adults suggest a reduced ability to generate rapid and coordinated postural responses to unexpected challenges. In line with previous evidence, age-related declines in sensory integration (François et al., 2011), neuromuscular function (Piirainen et al., 2010), and stimulus–response speed (Fabre and Lemaire, 2005) may underlie the compromised reactive balance control mechanisms, with cortical adaptations further modulating postural responses under challenging sensory conditions (Wang et al., 2025a).

In particular, the worsened postural responses observed, especially in the medio-lateral direction, may *reflect* additional compensatory strategies adopted by older adults to maintain stability when facing unexpected perturbations. Although the perturbation was delivered in the anterior–posterior direction, older adults exhibited increased medio-lateral oscillations, suggesting a loss of directional specificity in postural control. This may indicate a greater reliance on hip strategy compensation or a reduced ability of neural pathways to independently regulate lateral stability, which is critical for fall prevention (Wang et al., 2025b). Such decline in reactive postural adjustments may contribute to a higher susceptibility to balance loss in everyday situations (Bhatt and Pai, 2009; Oliveira et al., 2012; Zemková et al., 2016).

Moreover, the absence of significant differences in static balance performance, indicated by comparable Area95 values, aligns with previous findings (Peterka and Black, 1990; Gu et al., 1996; Liaw et al., 2009), suggesting preserved static balance performance in older adults. Nonetheless, the higher Mean Velocity in older adults reflects reduced postural control efficiency (Paillard and Noé, 2015). Thus, even though older adults retain static postural stability comparable to that of younger adults, such stability is sustained through a higher reliance on corrective adjustments. SampEn findings further contribute to this point by highlighting the role of postural control mechanisms. Specifically, SampEn is an index of CoP-trajectory regularity that reflects the automaticity (Roerdink et al., 2006) and adaptability (Anderson and Button, 2017) in postural regulation under steady-state conditions. The significant reduction in SampEn Y in older adults, compared to younger individuals, suggests a decrease in the automaticity of static balance control. Since SampEn Y refers to oscillations along the antero-posterior axis, the primary axis of human balance regulation (Kuo and Zajac, 1993; Winter, 1995), this finding indicates that aging may impair the ability to automatically regulate posture even during quiet standing. This suggests that, for older adults, maintaining stability under static conditions already relies on less automatic and more rigid control strategies.

Alternatively, the observed reduction in SampEn Y may also reflect an increased attentional involvement during quiet standing in older adults. Previous studies have shown that aging is associated with a greater allocation of attentional resources to postural control, even under steady-state conditions, as balance becomes less automated and requires more conscious regulation (Marsh and Geel, 2000). In this perspective, lower CoP complexity may indicate a more attention-driven control strategy rather than solely a loss of adaptability (Doyon et al., 1998; Donker et al., 2007). Furthermore, lower SampEn values generally reflected more regular and predictable sway dynamics, which are often related to increased postural rigidity and reduced adaptability (Donker et al., 2007; Roerdink et al., 2011). A greater regularity of the CoP trajectory suggests that older adults rely on more constrained and cognitively demanding control strategies (Geurts and Mulder, 1994; Melzer et al., 2001; Roerdink et al., 2006). Importantly, SampEn was computed only for static trials, due to the nonstationary nature of postural responses following perturbations. Therefore, the present findings do not allow for a direct quantification of automaticity during dynamic, perturbation-based tasks. This reduced automaticity may reflect a shift toward a more voluntary postural control and less flexible sensorimotor processes. This interpretation aligns with evidence that older adults often rely on more stereotyped postural patterns to maintain stability (Manor et al., 2010), with a shift toward more rigid and less variable control strategies as automaticity declines (Richer and Lajoie, 2020). From a physiological perspective, this reduction in complexity may indicate a diminished efficiency of the postural control system. Indeed, the reduced SampEn Y observed in older adults aligns with the group differences detected in Mean Velocity during static stance. The concomitant higher Mean Velocity and lower SampEn Y in older adults reflect a coherent age-related alteration in postural regulation: static balance performance in older adults is maintained, but at the cost of less efficiency, reduced automaticity, and greater conscious regulation.

#### Relationship between static and dynamic balance performance

Evidence regarding the relationship between static and dynamic balance is often inconsistent (Carter et al., 2002; Hrysomallis et al., 2006; Granacher et al., 2012; Sell, 2012), likely due to the complexity of postural control mechanisms and the diversity of balance tasks used in previous research. In contrast to earlier studies (Granacher et al., 2011; Muehlbauer et al., 2012) and in line with our previous work (Rizzato et al., 2025b), we used the same CoP-related parameters across both static and dynamic assessments, providing a more robust methodological framework. Nevertheless, no significant correlations between static and dynamic balance performances emerged, either within each age group and across the entire sample. Although a low correlation between static and dynamic Mean Velocity emerged in older adults, its small magnitude suggests that it may reflect a general decline in postural control efficiency with aging rather than a meaningful relationship between static and reactive balance mechanisms. This finding strengthened the task-specificity of balance performance (Rizzato et al., 2025a,b): although static and dynamic balance are regulated by the same neural structures (Takakusaki et al., 2016), they rely on distinct control mechanisms. Indeed, in static balance, the external forces challenging postural control are minimal, and the task is generally straightforward unless a system is impaired (Winter, 1995). Bipedal stance can be modeled as a single inverted pendulum (Morasso et al., 2019), with anterior–posterior oscillations primarily regulated at subcortical levels and modulated by local feedback loops (Nashner and McCollum, 1985). In contrast, perturbation-based tasks impose larger destabilizing forces and require rapid and coordinated postural adjustments to maintain stability. Reactive postural responses are primarily reflexive, allowing the body to respond quickly to unexpected changes in support or external forces (Horak and Nashner, 1986; Jacobs and Horak, 2007). These task-dependent differences highlight why static and dynamic balance assessments provide complementary information and support the need for separate evaluation protocols in both research and clinical settings.

This study presents some limitations that should be acknowledged. Although the perturbation-based protocol elicited unexpected balance challenges, only a single forward perturbation with fixed magnitude and velocity was applied. This choice allowed older participants to complete the trials without stepping, ensuring assessment of pure reactive balance, but it limits the generalizability of the findings to other directions, magnitudes, or more complex perturbations. Additionally, because the sample consisted solely of healthy younger and older adults, differences identified in this study may not be generalized to populations with balance impairments. Finally, habitual physical activity was not quantitatively assessed and cannot be fully excluded as a potential confounding factor; however, the dynamic task primarily elicits automatic, task-specific mechanisms, minimizing the influence of habitual physical activity.

## Conclusion

Overall, our findings highlight that age-related changes in balance are task dependent. Older adults showed preserved balance performance during quiet standing, although with reduced postural control efficiency. Furthermore, the lower complexity of CoP trajectories among older adults suggests the use of more rigid and cognitively demanding strategies to maintain stability in static standing. In contrast, clear impairments occurred in dynamic, perturbation-based tasks. These were characterized by reduced reactive postural responses and lower postural control efficiency, indicating poorer compensatory adjustments to unexpected balance challenges. These findings reinforce the notion that static measures alone may underestimate age-related decrement of balance regulation. Importantly, our results also showed that static and dynamic balance performance are not related, underscoring the need for separate testing procedures for static and dynamic balance in both research and clinical settings. Such distinction allows for more accurate assessments of postural control and supports the development of interventions tailored to the specific challenges induced by different types of balance tasks.

## Data Availability

The raw data supporting the conclusions of this article will be made available by the authors, without undue reservation.
